# Lithium Niobate – Enhanced Photoacoustic Spectroscopy

**DOI:** 10.1016/j.pacs.2023.100577

**Published:** 2023-11-30

**Authors:** Aldo F.P. Cantatore, Giansergio Menduni, Andrea Zifarelli, Pietro Patimisco, Miguel Gonzalez, Huseyin R. Seren, Vincenzo Spagnolo, Angelo Sampaolo

**Affiliations:** PolySense Lab, Dipartimento Interateneo di Fisica, University and Politecnico of Bari, Via Amendola 173, Bari 70126, Italy; PolySense Lab, Dipartimento Interateneo di Fisica, University and Politecnico of Bari, Via Amendola 173, Bari 70126, Italy; PolySense Innovations srl, Via Amendola 173, Bari 70126, Italy; Aramco Services Company, 17155 Park Row, Houston, TX 77084, USA; PolySense Lab, Dipartimento Interateneo di Fisica, University and Politecnico of Bari, Via Amendola 173, Bari 70126, Italy; PolySense Innovations srl, Via Amendola 173, Bari 70126, Italy

**Keywords:** Lithium niobate forks, LiNTF, Lithium niobate-enhanced photoacoustic spectroscopy, LiNPAS, QEPAS, Measurement of fluid properties, High quality factors resonators, Integrated photonic devices for gas sensing

## Abstract

In this work, we report on the novel employment of lithium niobate tuning forks as acoustic transducers in photoacoustic spectroscopy for gas sensing. The lithium niobate tuning fork (LiNTF) exhibits a fundamental resonance frequency of 39196.6 Hz and a quality factor Q = 5900 at atmospheric pressure. The possibility to operate the LiNTF as a photoacoustic wave detector was demonstrated targeting a water vapor absorption line falling at 7181.14 cm^−1^ (1.39 µm). A noise equivalent concentration of 2 ppm was reached with a signal integration time of 20 s. These preliminary results open the path towards integrated photonic devices for gas sensing with LiNTF-based detectors on lithium niobate platforms.

In the last couple of decades, lithium niobate (LiN) has arisen as one of the most employed materials in the integrated photonics field exploiting its strong electro-optic, acousto-optic and nonlinear optical properties [1], [2] combined with a high refractive index, stable physical and chemical properties and a wide transparency spectral window (0.4 – 5 µm) [3], [4]. These features boosted a rapid development of LiN on insulator technology, consisting of LiN thin films bonded through a buried oxide layer to a LiN or silicon substrate, allowing the realization of basic structures, such as optical waveguides and resonant cavities [4], as well as the integration of LiN acousto-optic modulators based on a racetrack resonator [5], and electro-optic modulators based on Mach-Zender interferometers with integrated electrodes, which have been extensively developed throughout the last twenty years [6], [7], [8]. Because of its large electromechanical coupling coefficient, LiN has been also widely used for the development of various piezoelectric devices, including tuning forks (TFs), filters, transducers, actuators, and sensors [9], [10]. For example, LiN tuning forks (LiNTFs) have already been largely employed as viscosity and density sensors for fluid properties measurements [11], [12], [13], [14]. Moreover, it is well known that TFs can be used as sound wave detectors. This application created a perfect breeding ground in Quartz-Enhanced Photoacoustic Spectroscopy (QEPAS) for gas sensing. In QEPAS, a quartz tuning fork (QTF) is employed for detecting weak sound waves generated via photoacoustic effect within a gas sample as a consequence of non-radiative energy relaxation induced by infrared modulated light absorption [15], [16].

In this work we explore the possibility to employ a LiNTF as a piezoelectric transducer in photoacoustic spectroscopy for sound wave detection. For ease of reading, in the following the technique will be referred to as Lithium Niobate-enhanced Photoacoustic Spectroscopy (LiNPAS). The strain and the electric field distribution when the LiNTF is excited at its fundamental in-plane flexural mode are modelled with a Finite Element Analysis. Using the simulation results as reference, the LiNTF prototype is employed in a LiNPAS sensor for water vapor detection.

As already demonstrated in QEPAS, the fundamental TF in-plane anti-symmetric flexural mode can be efficiently excited by focusing the laser beam through the TF prongs, close to the vibration antinode [17]. In this way, the prongs are put into oscillation by the acoustic wave generated via photoacoustic effect impacting on the internal surface of prongs. A finite element method (FEM) simulation of the LiNTF fundamental in-plane anti-symmetric flexural mode was performed using COMSOL Multiphysics. The geometry of the LiNTF roughly mimics the standard 32.7 kHz-QTF employed in a QEPAS sensor for the first time in 2002 [18]. With respect to the quartz crystal, the effective piezoelectric coefficient d_23_ of 128° y-cut LiN, which is the only one involved in the excitation of the fundamental in-plane anti-symmetric flexural mode, is nearly 10 times higher than d_11_ of α-quartz (2–3 pC/N) [14], [19]. Moreover, LiN has higher density and Young’s modulus (4650 kg/m^3^ and 145 GPa, respectively) with respect to quartz (2660 kg/m^3^ and 72 GPa). The simulated LiNTF geometry is sketched in Fig. 1a: the prongs have a length of 3.18 mm, a width of 0.45 mm, and a thickness of 1.25 mm, with a spacing of 0.35 mm.

**Fig. 1:**
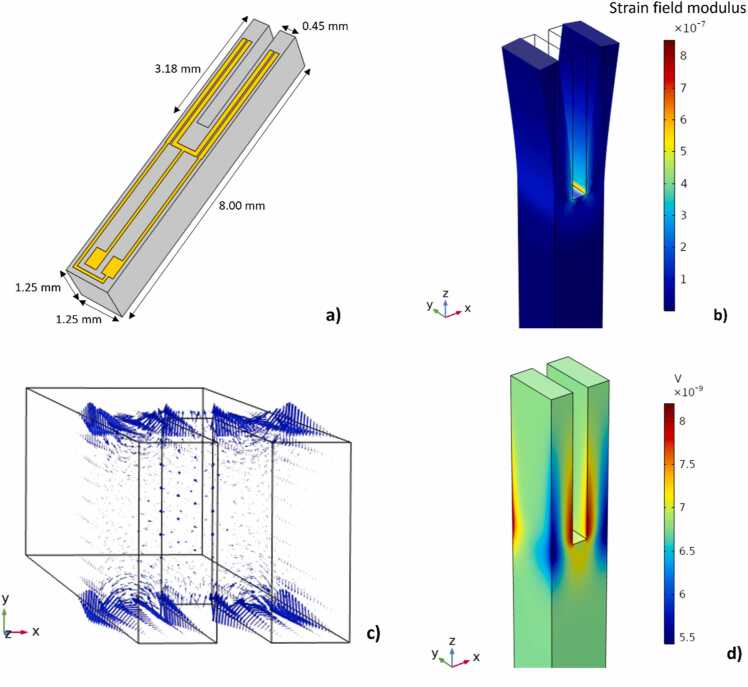
a) Geometry and gold pad scheme of the employed LiNTF; b) COMSOL simulation of the displacement of each prong when excited at the fundamental in-plane anti-symmetric flexural mode. The 128° y-cut was selected for the material. The strain field modulus distribution is represented in the colour scale bar. A resonance frequency of 39182.0 Hz is predicted; c) COMSOL simulation of the electric field generated within each prong via piezoelectric effect (blue arrows); d) COMSOL simulation of the electric potential generated within the TF via piezoelectric effect.

The simulation of the mechanical behavior of the LiNTF was performed employing the Solid Mechanics module. A resonance frequency f_0_ = 39182.0 Hz was predicted by means of an eigenfrequency study and used as a reference value for the characterization of the frequency response of the LiNTF. Then, employing the Electrostatics module and coupling it to the Solid Mechanics one by means of the Piezoelectric Effect interface module, the following equations were solved by the FEM software within the LiNTF volume:

(1) E=−∇V

(2) $$\nabla ∙D=\rho_{V}$$

(3) $$D=\epsilon_{0}\epsilon_{\mathrm{LiN}}E+P_{\mathrm{pze}}$$

where E*,* V, and D are the electric field, the electric potential, and the Maxwell displacement field, respectively, generated via piezoelectric effect within the LiNTF, $\rho_{V}$ is the volume density of piezoelectric charge, $\epsilon_{\mathrm{LiN}}$ is the 128° y-cut LiN relative permittivity tensor, and $P_{\mathrm{pze}}$ is the piezoelectric polarization density field. The results of the simulation are shown in Fig. 1b-d. The strain field (Fig. 1b) is mainly localized on the internal lateral surface of the two prongs, close to the clamped end. The electric field (Fig. 1c) is spatially distributed along the front surface of TF prongs with its intensity decreasing moving away from their surface. The electric potential distribution on the TF surface is displayed in Fig. 1d, with the piezoelectric charge being distributed accordingly. Following the simulation results, a LiNTF was fabricated in collaboration with TE Connectivity Ltd. on a 128° y-cut LiN wafer. Two pairs of gold electrodes, having the geometry depicted in Fig. 1a, are deposited on one of the LiNTF front surfaces to collect the generated piezoelectric charges. The electrode layout matches the polarity of the electric potential as well as it covers surface zones where the electric potential reaches maximum intensity (Fig. 1a to be compared with Fig. 1d), in order to maximize the charge collection efficiency.

The realized LiNTF was mounted as a photoacoustic detector in a LiNPAS sensor depicted in Fig. 2.

**Fig. 2:**
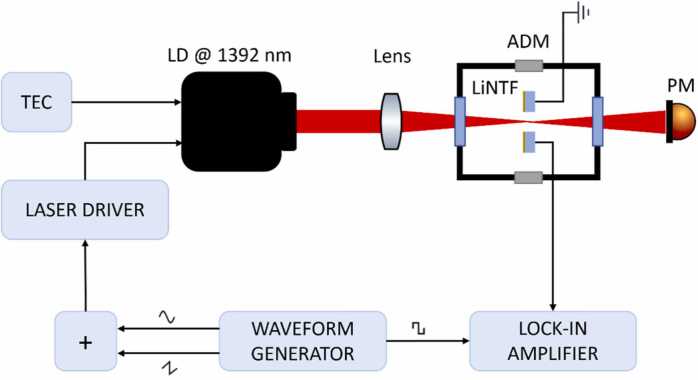
Schematic of the experimental apparatus. Black arrows represent electronic connections. TEC – ThermoElectric Cooler, LD – Laser Diode, LiNTF – Lithium Niobate Tuning Fork, ADM – Acoustic Detection Module, PM – Power Meter.

As a proof-of-concept, four water vapor absorption lines in the 7180.54–7190.00 cm^−1^ wavenumber range [20] falling at 7181.15 cm^−1^, 7182.21 cm^−1^, 7182.94 cm^−1^ and 7185.60 cm^−1^, respectively, were targeted employing a Nanoplus laser diode (LD). The LD central emission wavelength was 1392 nm with an output power of 10 mW. The light source was controlled by means of a Thorlabs LDC202C current driver and a Thorlabs TED200C temperature controller, operating it at T = 30 °C. A lens having a 40 mm focal length was used to focus the laser beam between the prongs of the LiNTF, mounted in an acoustic detection module (ADM). All the measurements were carried out in static conditions with the ADM isolated from the environment and filled at atmospheric pressure with a gas sample of standard air, composed of 20% O_2_, 1.2% H_2_O and 78.8% N_2_.

First, the LiNTF response in a frequency range containing the fundamental mode resonance was obtained for determining both the resonance frequency and the quality factor of the resonator. The laser emission wavelength was locked at the strongest H_2_O absorption line in the LD tuning range, located at 7181.15 cm^−1^ with a linestrength of 1.53 ∙ 10^−20^ cm/molecule [20]. The characterization was performed in wavelength modulation and first harmonic detection (WM-1 f): the LD current was sinusoidally modulated, and the LiNTF signal demodulated at the same excitation frequency by a lock-in amplifier (Zurich Instruments MFLI). To reconstruct the LiNTF response curve, the LD current modulation frequency was varied step-by-step in the range 39150–39245 Hz. The frequency response of the LiNTF exhibits a resonance close to the simulated fundamental mode and it is shown in Fig. 3 (datapoints).

**Fig. 3:**
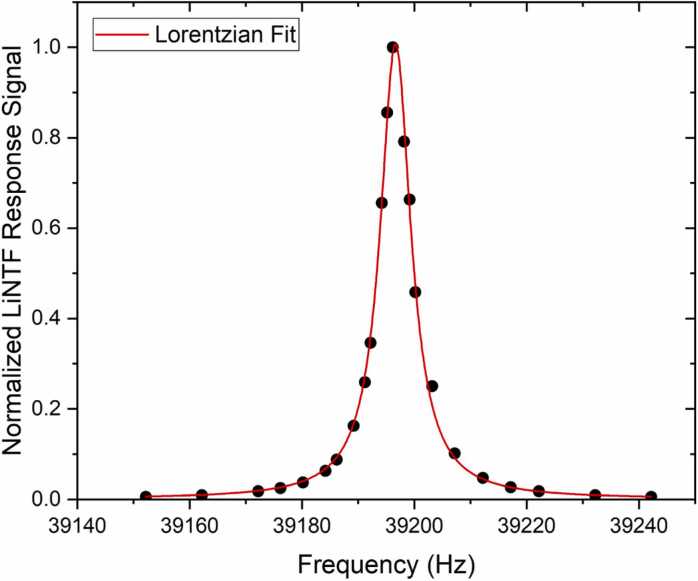
Normalized resonance curve of the LiNTF (black dots). A fundamental resonance frequency of f_0_ = 39196.6 Hz and a quality factor of Q = 5900 were extracted at atmospheric pressure using a Lorentzian fit (red curve).

A resonance frequency f_0_ = 39196.6 Hz and a quality factor Q = 5900 were estimated performing a Lorentzian fit (red solid line in Fig. 3) of the datapoints, resulting in a good agreement with the resonance frequency predicted by the FEM model, with a discrepancy of 0.34‰.

LiNPAS measurements were carried out in the wavelength modulation and second harmonic detection (WM-2f) scheme [21]. Therefore, the laser driver current was modulated applying a sinewave at half of the LiNTF resonance frequency, while the f_0_ component of the generated LiNTF signal was extracted by the lock-in amplifier. A slow 2.5 mHz sawtooth ramp was superimposed to the sinusoidal modulation to scan the whole tuning range of the laser (see Fig. 2). The acquired 2 f-LiNPAS signal is shown in the upper panel of Fig. 4, while the whole absorption spectrum of water vapor in a matrix of standard air is displayed in terms of the absorption coefficient in the lower panel of the same graph.

**Fig. 4:**
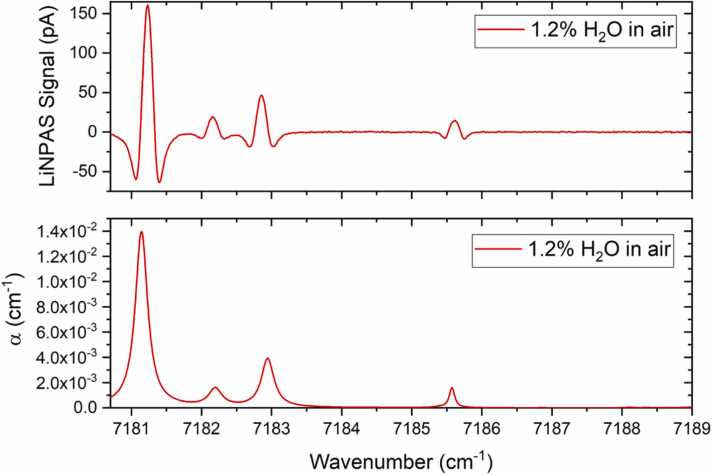
In the upper panel, 2 f-LiNPAS spectrum measured for a sample of air with a 1.2% concentration of absolute humidity at atmospheric pressure. In the lower panel the absorption coefficient for a standard air sample from HITRAN database.

As predicted, four water vapor peaks were detected within the LD tuning range, with a maximum signal of 161.3 pA corresponding to the water absorption peak at 7181.14 cm^−1^. The noise level was evaluated as the 1σ-standard deviation of the acquired data in the 7188.00 – 7189.00 cm^−1^ range, far from H_2_O absorption features. Considering a noise of 0.4 pA, the signal to noise ratio (SNR) for the most intense peak is ∼400, corresponding to a noise equivalent concentration (NEC) of ∼30 ppm at 100 ms integration time and a normalized noise equivalent absorption (NNEA) of 2.7 ∙ 10^−7^ Wcm^−1^Hz^−1/2^ [22].

The performances of the LiNTF prototype were compared to those of a standard 32 kHz QTF, which has both similar geometry, and resonance frequency (as shown in Fig. S1). The measured resonance frequency and quality factor at atmospheric pressure of the employed QTF are f_0_ = 32739.9 Hz and Q = 9100, respectively. The QTF frequency response is shown in Fig. S1b of supplementary material. Replacing the LiNTF with the selected QTF in the setup shown in Fig. 2 and working in the same experimental conditions of the LiNPAS measurements, a 2 f-QEPAS spectrum of the standard air sample was acquired and it is reported in Fig. S2 of supplementary material. Considering a noise of 0.9 pA, the SNR for the most intense peak is ∼450 at 1.2% water vapor concentration, corresponding to a NEC of ∼26 ppm at 100 ms integration time and a NNEA = 2.4 ∙ 10^−7^ Wcm^−1^Hz^−1/2^. These results show that the LiNTF prototype performances are comparable to those of a standard QTF.

Finally, an Allan – Werle deviation analysis was performed to determine the noise dependence on the lock-in integration time [23]. For this analysis, the laser wavelength was locked at 7184.30 cm^−1^, where no absorption occurs, working in the WM-2 f scheme. The Allan – Werle deviation analysis of the acquired signal is shown in Fig. 5. For an integration time of 20 s, a noise reduction of one order of magnitude is achieved, down to 0.03 pA, corresponding to a NEC of ∼2 ppm. The detection limit, mainly depending on the thermal noise reduction at increasing integration times, scales down as 1/√t just like standard QTFs in QEPAS [22], [23].

**Fig. 5:**
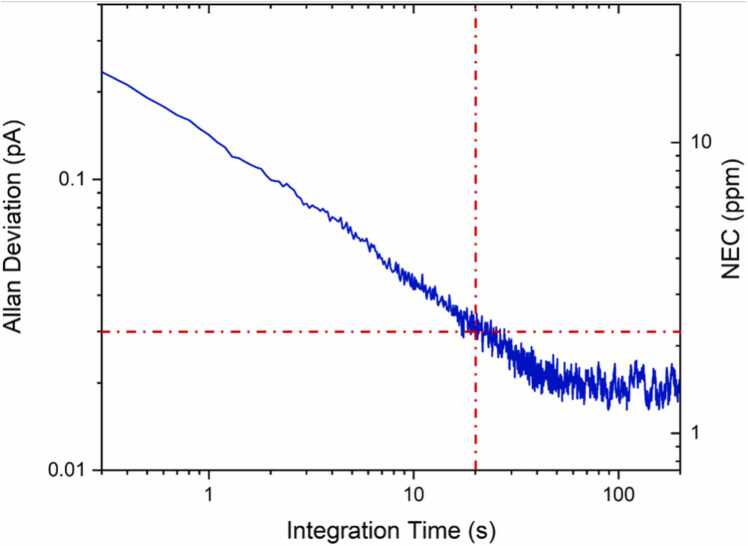
Allan-Werle deviation analysis performed over a two hour-long acquisition of the LiNPAS signal. On the right y axis, the NEC associated to the noise level is indicated. An increase up to 20 s of the integration time allows for a noise reduction by one order of magnitude.

In conclusion, the possibility to employ a lithium niobate tuning fork resonator as a transducer in photoacoustic spectroscopy-based gas sensors was demonstrated. Water vapor was selected as the target gas, achieving an SNR of 400 for a 1.2% concentration of H_2_O at atmospheric pressure and 100 ms lock-in integration time. An Allan – Werle deviation analysis showed that a noise reduction of one order of magnitude can be achieved increasing the integration time up to 20 s. The LiNTF showed comparable performances with respect to a standard QTF employed as a photoacoustic detector under the same experimental conditions. These results set a promising starting point for the development of fully integrated LiNPAS-based trace gas sensors on LiN substrates. In order to improve the LiNTF performances as piezoelectric transducers in photoacoustic spectroscopy, two main strategies will be adopted: i) the coupling with a pair of acoustic resonator tubes, which has been proved to provide SNR enhancement factors starting from × 30 up to ∼× 60 for QTFs [24] and ii) the design and test of new LiNTF geometries, comparing their performances to find the most suitable one for photoacoustic detection. Furthermore, the performances of LiNTFs as light detectors in a light-induced thermo-elastic spectroscopy (LITES) configuration can be also investigated for the development of tunable diode laser absorption spectroscopy (TDLAS)-based gas sensors [25], [26], [27], [28].
