# Supplementary figures and images for: Lithium Niobate – Enhanced Photoacoustic Spectroscopy
Source: Photoacoustics. 2023 Nov 30;35:100577. doi: 10.1016/j.pacs.2023.100577 (PMC10749900; doi:10.1016/j.pacs.2023.100577)

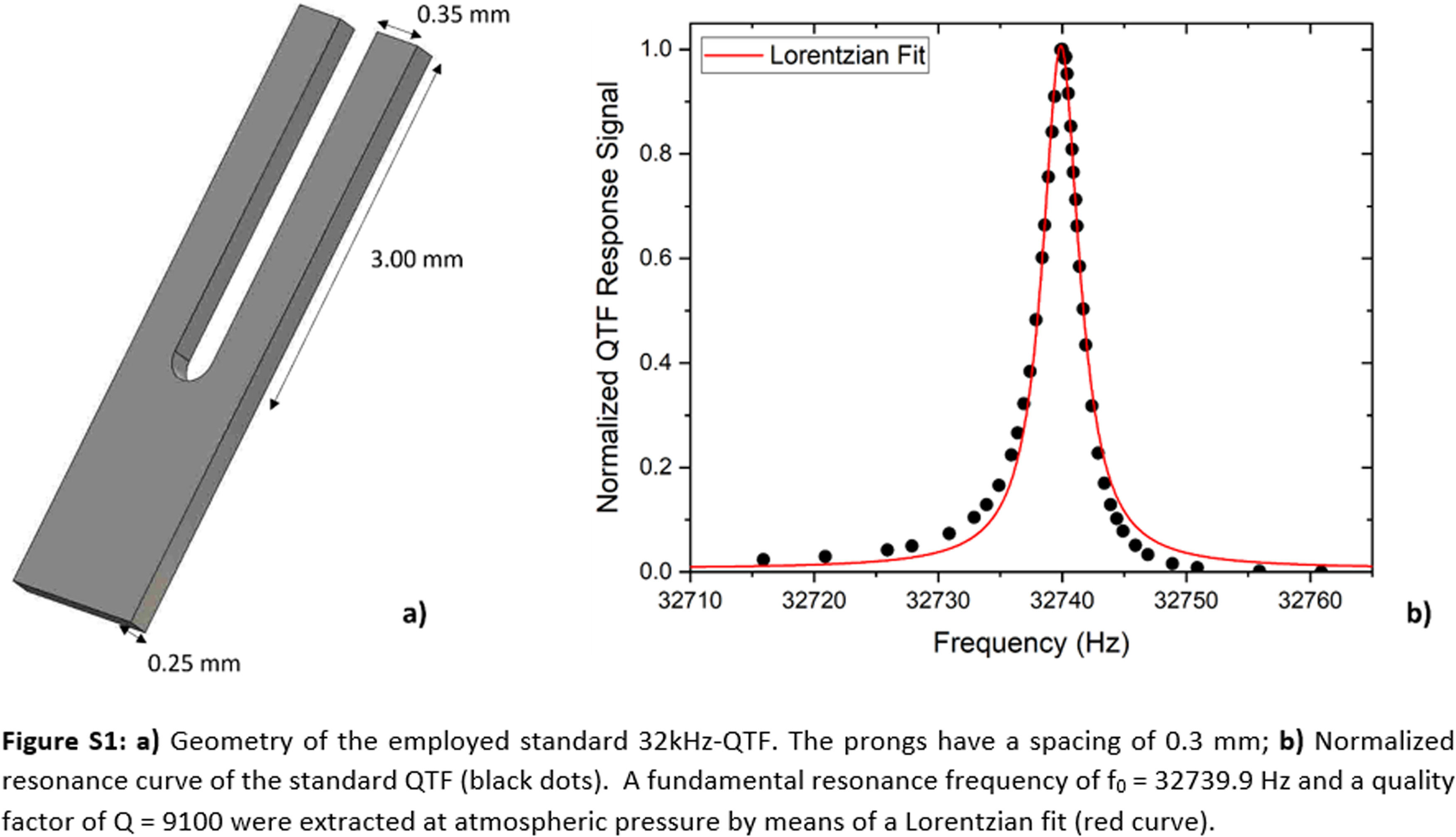

Supplement: Supplementary file 1 — Supplementary material [file mmc1.jpg]

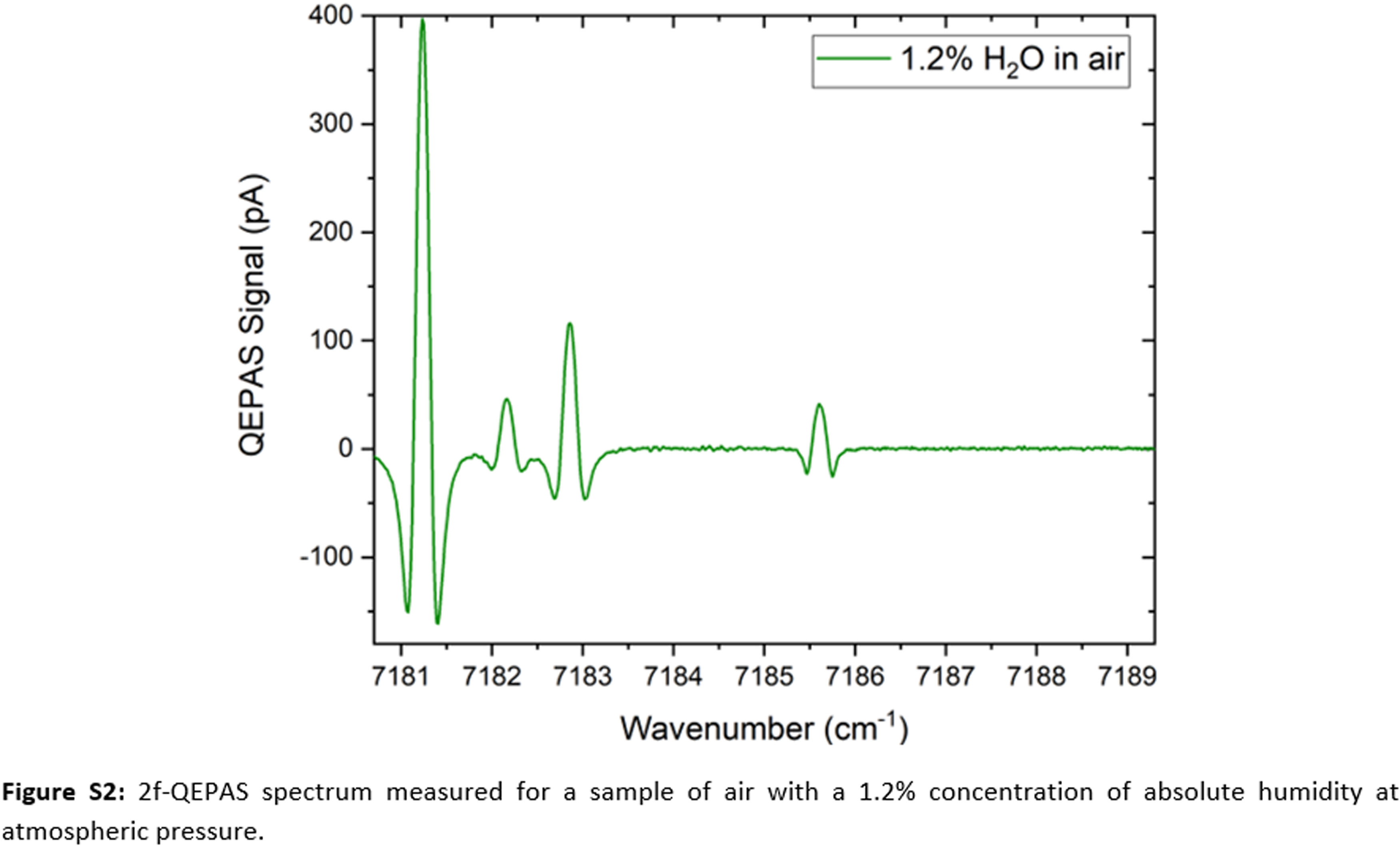

Supplement: Supplementary file 2 — Supplementary material [file mmc2.jpg]
